# Artificial intelligence-based triage of large bowel biopsies can improve workflow

**DOI:** 10.1016/j.jpi.2022.100181

**Published:** 2023-01-02

**Authors:** Frederick George Mayall, Mark David Goodhead, Louis de Mendonça, Sarah Eleanor Brownlie, Azka Anees, Stephen Perring

**Affiliations:** a Department of Cellular Pathology, Musgrove Park Hospital, Taunton TA1 5DA, UK; b Longshot Systems, 199 Chiltern Court, London NW1 5SD, UK; c Diagnostic Path Solutions, 12 North Bar, Banbury OX16 0TB, UK

**Keywords:** Digital pathology, Information technology, Cancer diagnosis, Artificial intelligence, Colon, Rectum, Large bowel, Workflow, Adenocarcinoma, Adenoma

## Abstract

**Background:**

Large bowel biopsies are one of the commonest types of biopsy specimen. We describe a service evaluation study to test the feasibility of using artificial intelligence (AI) to triage large bowel biopsies from a reporting backlog and prioritize those that require more urgent reporting.

**Methods:**

The pathway was developed in the UK by National Health Service (NHS) laboratory staff working in a medium-sized general hospital.   The AI platform was interfaced with the slide scanner software and the reporting platform’s software, so that pathologists could correct the AI label and reinforce the training set as they reported the cases.

**Results:**

he AI classifier achieved a sensitivity of 97.56% and specificity of 93.02% for the case-level-diagnosis of neoplasia (adenoma and adenocarcinoma) and for an AI diagnosis of any significant pathology (i.e., adenomas, adenocarcinomas, inflammation, hyperplastic polyps, and sessile serrated lesions) sensitivity was 95.65% and specificity 92.96%. The automated AI diagnostic classification pathway took approximately 175 s per slide to download and process the scanned whole slide image (WSI) and return an AI diagnostic classification. Biopsies with an AI diagnosis of neoplasia or inflammation were prioritized for reporting while the remainder followed the routine reporting pathway. The AI triaged pathway resulted in a significantly shorter reporting turnaround time for pathologist verified neoplastic cases (P < 0.001) and inflammation (P < 0.05). The project’s costs amounted to  £14800, excluding laboratory staff salaries. More time and resources were spent on developing the interface between the AI platform and laboratory IT systems than on the development of the AI platform itself.

**Conclusions:**

NHS laboratory staff were able to implement an AI solution to accurately triage large bowel biopsies into several diagnostic classes and this improved reporting turnaround times for cases with neoplasia or with inflammation.

## Background

Large bowel biopsies are one of the commonest types of biopsy specimens to be submitted to cellular pathology laboratories in the UK. A small number of these, less than 5%, are taken from lesions that are clinically obvious carcinomas and these are usually submitted for urgent reporting. The remainder are mainly a mixture of various forms of polyp and inflammatory lesions, together with a significant proportion, approximately 60%, showing no abnormality. Biopsies that are not thought to have arisen from a carcinoma, or similar sinister lesion, are usually given a relatively low priority for reporting and in an understaffed department, they will often be allocated to the reporting backlog or be sent to an external service provider for remote reporting. This can lead to reporting being delayed for several weeks. This delay is undesirable for the management of the patient and would be non-compliant with some of the key performance targets of NHS pathology laboratories. Nevertheless, such delays are quite common for gastrointestinal biopsies and non-urgent biopsies from other sites.

**Fig. 1:**
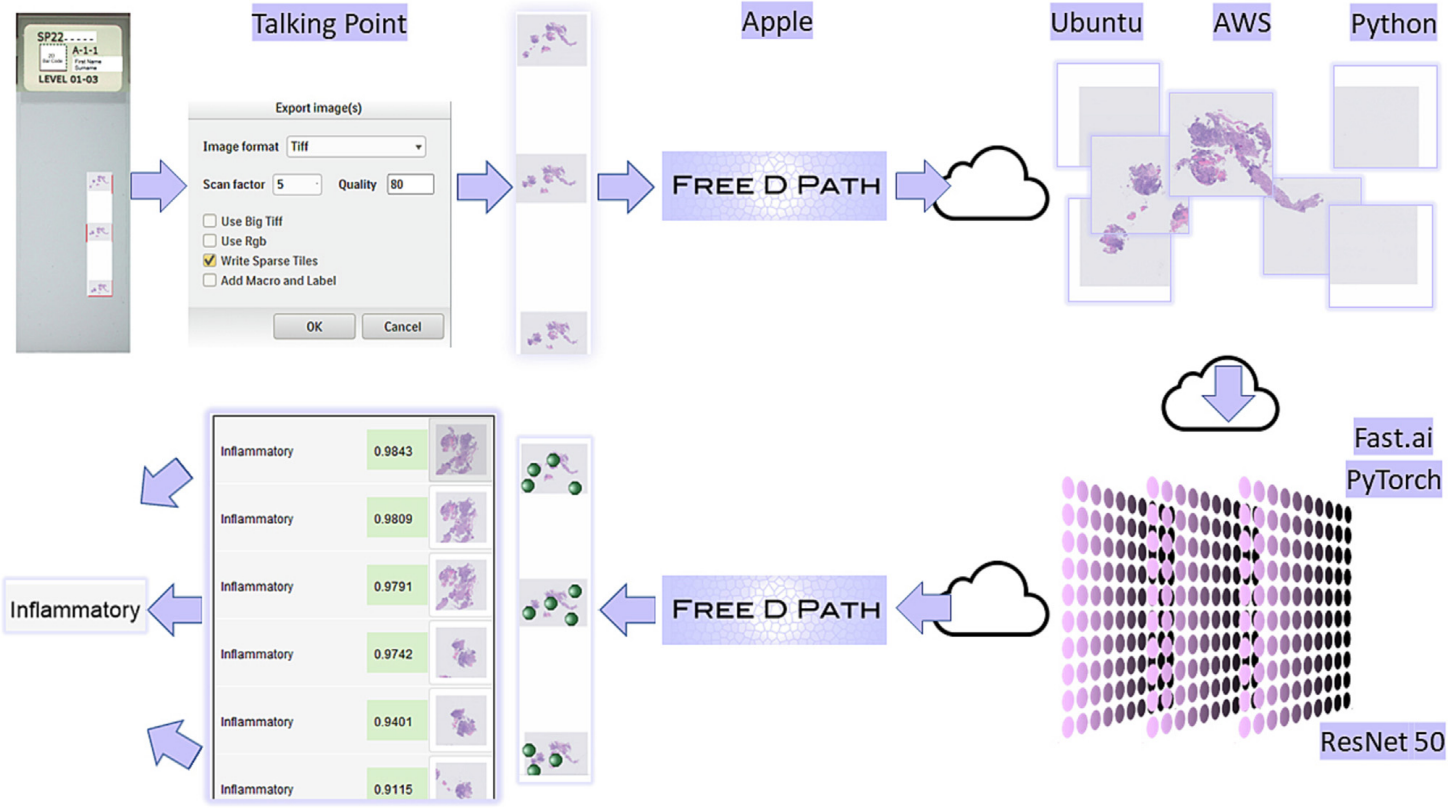
The scanned slide images were downloaded as a TIFF file at scan factor 5, imported into the Free D Path reporting software, sent to a virtual machine for more image processing and tiling. The tiles were submitted for classification and the tile images along with diagnostic labels were sent back to the reporting software and this software calculated a final AI classification for the case. This was used to decide the reporting priority for the case. The software also displayed the tiles to the reporting pathologist so they could be reviewed during reporting of the case and corrected if needed. Corrected tiles were automatically flagged for the next round of retraining. Various proprietary and open-source software solutions were used including Talking Point, Apple (FileMaker), Ubuntu, AWS, Python (including Scikit-Image), PyTorch, Fast.ai, and various established laboratory systems including Philips IMS for slide scanning and Cerebro (Leica Biosystems) for laboratory pathway management.

When a patient has an endoscopic examination of the large bowel, the operator may take several mucosal biopsy specimens from different sites in the large bowel even if no endoscopic abnormality is seen. Typically, one to five biopsy specimens are taken during a single endoscopic examination, but sometimes more than 10. These specimens are sent to the laboratory in separate formalin containers with a single request form describing the biopsy sites, and a case is created on the laboratory IT system and given an alphanumeric case number, for example SP2312345. The individual specimens that make up the case are then assigned specimen identifiers, for example “SP2312345-A, SP2312345-B etc. and then each specimen may generate several slides, designated SP2312345-A-1, SP2312345-A-2 etc. Thus, a case may generate many slides each with its own diagnostic classification, but the pathologist will report all the slides at the same time and issue a single report for the case as a whole. In addition, it is usual for most types of small biopsy, including large bowel biopsies, to have at least three levels cut from the paraffin block for examination. Usually, all three levels can be placed on a single glass slide and consequently a large bowel biopsy WSI will usually include three sections of tissue that are similar (Fig. 1).

The processing of biopsies to create scanned WSIs has been comprehensively described in a previous paper.^1^ Most large bowel biopsies consist mainly of large bowel mucosa and lamina propria, with a little superficial submucosal tissue. However, excision biopsies of pedunculated polyps can contain abundant smooth muscle and fibrous stroma. Small bowel from the ileocaecal junction is often included in the biopsies. Low rectal biopsies sometimes include some anal squamous mucosa. The biopsy tissue sections may show a variety of artifacts including fecal material, diathermy artifact, crush artifact, and folded tissue sections. The WSI may include features other than the biopsy tissue. There may be air bubbles in the coverslip mountant, misaligned coverslip margins, dust on the cover slip, text printed on the glass or stuck-on paper labels, and other artifacts unrelated to the biopsy tissue. These artifacts and extraneous features have the potential to confuse AI classification.

There are usually multiple different information systems involved in the reporting pathway of cellular pathology specimens. Typically, there will be a laboratory information system in which a case is first created. In the UK, this is often an antiquated database that is shared with other pathology specialties such as biochemistry, hematology, and microbiology. Often this will be interfaced with some specialist cellular pathology process management software, such as the Cerebro platform (Leica Biosystems), and this in turn will be interfaced with proprietary software that supports specialist equipment including immunostainers and slide scanners. Usually, there will also be another specialist cellular pathology reporting platform on which the pathologist will create their report and this report will then be sent to a patient information system to be integrated into the patient’s electronic clinical record. It is widely recognized that interfacing with these platforms can be challenging, and the integration of an AI classification platform is not a simple proposition.

AI-based image classifier platforms are now widely available and have become relatively easy to use with conventional digital photos. The classification of WSIs is a more challenging problem. This is mainly due to their enormous size, typically greater than   60000 pixels wide and high or more than 200 times larger than the type of image typically taken on a modern smartphone. In these images, a pixel is about 0.25 μm across. This is 10 times smaller than the size of a red blood cell. These WSIs generate similarly enormous files, typically greater than 250 MB as a pyramidal TIFF and sometimes in excess of 2 GB. Another peculiar feature is their pyramidal structure. The file is a pyramid of multiple image layers, typically nine or more, with graduated levels of resolution. This structure has the advantage of being quicker to navigate when used with specialist viewing software. When the amount of tissue on the slide is small, as it tends to be for large bowel biopsies, then the size of the image file is reduced. Even so, it is still not possible to submit the whole image for AI classification. The main constraint is the memory of the graphics-processing unit. Instead, the images need to be downsized, for example from the equivalent of a ×40 objective image to ×5, and then segmented into square tiles, typically 256 × 256 pixels or 512 × 512 pixels (Fig. 1). Information is lost in this process but the interpretation of some types of biopsies, including large bowel biopsies, depends more on the low-power features than high-power features. This approach would typically generate between 10 and 50 tiles of 512 × 512 pixels per scanned slide image once empty space has been removed from the image. These tiles can be submitted for AI training and classification. There are some open-source Python libraries that have been widely adopted to support this WSI processing pathway. There are several cloud-based virtual machine services that can allow users to process WSIs, and to train and use an AI-based image classifier. For this type of image classification problem, a Residual Network (ResNet) is usually used. This is a form of convolutional neural network (CNN) architecture that has become widely adopted for image analysis tasks.

There have been previous attempts to use AI to interpret digitized diagnostic large bowel biopsies.^2^ Some of these have demonstrated good diagnostic sensitivity and specificity in a research setting.^2^^,^^3^ However, there are few published examples of artificial intelligence in operation in a real-life cellular pathology laboratory, particularly for the classification of large bowel biopsies. Advances in this field have primarily been led by academic data scientists, and the mundane and time-consuming problem of integrating the AI into a live diagnostic laboratory work stream has not been the main focus of their interest. However, with the improved usability of AI platforms it is now potentially feasible for diagnostic laboratory staff to use AI to create a “lab-developed” diagnostic classifier and integrate it with their specimen management and reporting software. This is what was attempted in our project.

## Method

This was a service evaluation study conducted in a medium-sized general hospital operated by the NHS in the UK to test the feasibility of using AI to triage large bowel biopsies that had accumulated in a reporting backlog, and to prioritize those that required more urgent attention. Our approach was to use AI to classify each slide and then to use an algorithm to classify the case as a whole and decide its priority for human pathologist reporting.

**Fig. 2:**
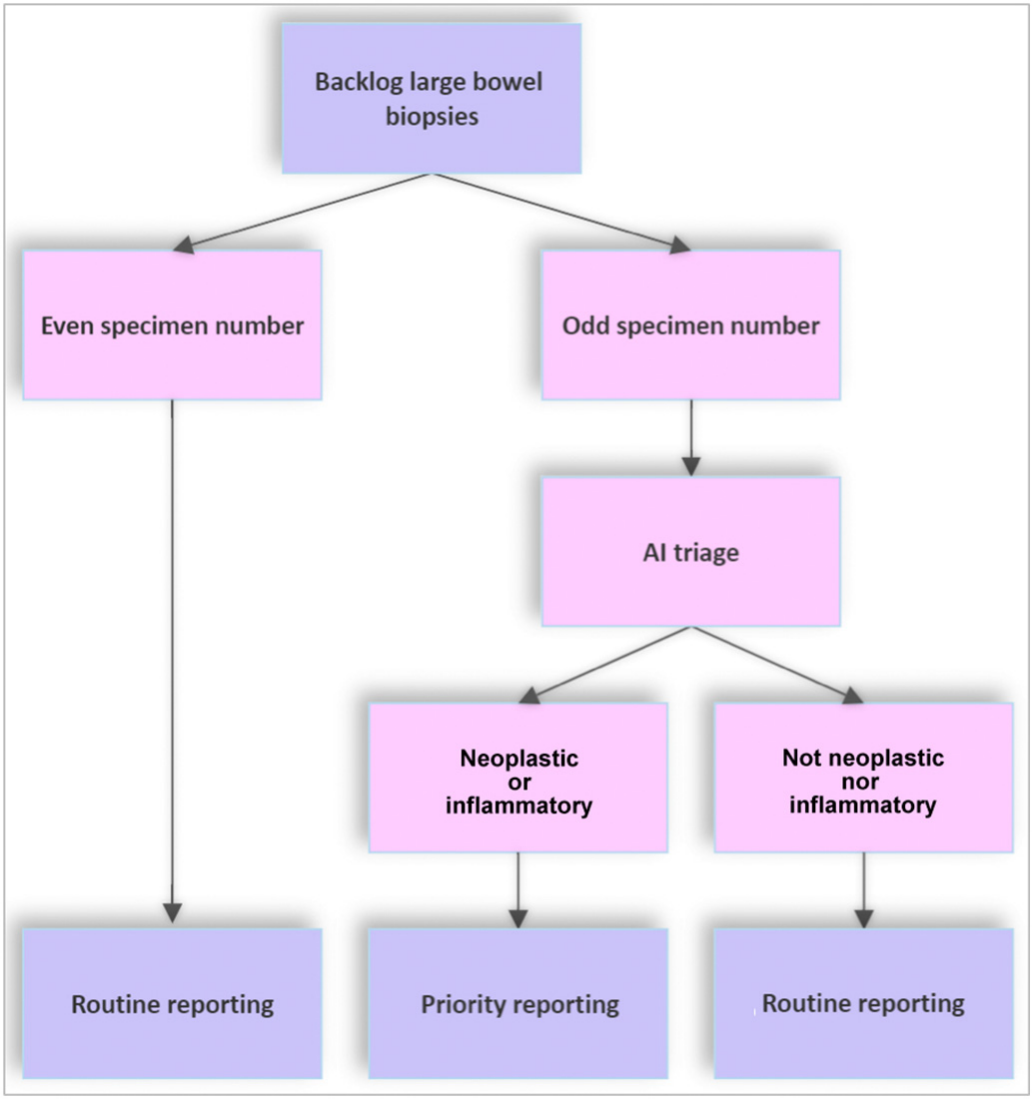
Case selection for the AI pathway was based on the numerical component of the specimen number.

In order to compare the performance of the standard reporting pathway with an AI augmented pathway, the backlog large bowel biopsy cases were separated according to odd or even case numbers, with odd numbers being allocated to the AI pathway and even numbers being allocated to the pre-existing routine pathway (Fig. 2). The slides of all cases were digitally scanned, according to the usual process for the laboratory, using a Philips IntelliSite Ultra Fast Scanner at ×40 objective magnification.

### Expert assistance

The laboratory had no academic staff and no pre-existing in-house AI expertise but there was remote support (from MDG) for image processing operations and machine learning carried out on the AWS virtual machine, and the laboratory had a pre-existing commercial relationship with Talking Point GHG that was used to procure the robotic process automation solution.

### Automated image download

Each day, usually in the evening, a list of newly scanned large bowel biopsy backlog cases with odd specimen numbers was created as a text (.txt) file on the laboratory’s IT network. The Philips scanner platform did not have an automated batch download function. It was possible to manually download images slide-by-slide, but this required many navigation steps and pauses for processing for each slide. We were able to replace this laborious task with a third-party robotic process automation method using Talking PointGHG software. This read the slide numbers from the text file and then imitated a high-speed human operator creating the images in a local network folder, labeled according to their slide names. The Philips digital pathology platform offered the option to downscale the images prior to download, and a “scan factor” of 5 was selected, effectively rendering a ×40 magnification image into a ×5 image. The platform also allowed an image “quality” option to be selected and this was set at 80 out of 100. The “write sparse tiles” option was also selected. This option produced a partially cropped image that partly eliminated areas of the slide in which there was no tissue (Fig. 1). These selections were made during the operation of the robotic process automation.

### Image processing pathway

As described above, the images were downscaled and cropped before being downloaded. They were then automatically imported from the shared drive into the laboratory's Free D Path reporting platform, developed using FileMaker (Apple Inc.). Once Free D Path had imported all of the images, it posted them to an Amazon Web Services (Amazon.com Inc.) virtual machine instance for further augmentation and then classification. First, the image was gray-scaled and inverted, and a Laplacian of Gaussian tissue detection method^4^^,^^5^ was applied. The tissue-rich areas were segmented into square tiles 512 × 512 pixels. No color normalization was used. Each tile was saved as a PNG file and then submitted for classification. Those slides in the AI pathway were processed and classified. No cases were rejected on the basis of there being too little tissue in the tiles.

### Training of the neural network

The method was developed iteratively over a period of approximately a year in 2020 and 2021.

The initial training set was created by a consultant pathologist (FM) labeling a set of 2281 image tiles derived from a variety of biopsies. This training set was used to optimize the AI. Then, this early iteration of the method was integrated into the diagnostic work stream and used to classify cases. The image-processing pathway was controlled by the laboratory’s Free D Path reporting software and the diagnostic predictions were returned to this software, so that the AI diagnoses for each case could be displayed in the pathologist’s reporting window. The pathologist could then easily re-label any tiles that were incorrectly labeled by the AI in about 5 s. These reclassified tiles were added to reinforce the training set, and every few weeks the network was retrained. Over time, the performance of the network improved as additional classification labels were added and the training set grew.

In initial trials, there were only three diagnostic classifications; normal, neoplastic, and inflammatory but other classifications were added as it became evident that they were required. Most WSIs include some artifact as outlined above, non-neoplastic polyps were seen in approximately 15% of slides and approximately 4% of slides included small bowel from the terminal ileum or an anastomosis site. Cases occasionally included anal mucosa, large sheets of connective tissue, or immunohistochemically stained tissue. The classifications used in the study are set out in Table 1.

**Table 1:** The diagnostic classes that were used and the number of training tiles for each. Pathologists were easily able to relabel tiles while reporting, and these tiles were added to the training set. Retraining was undertaken every few weeks, depending on how many new tiles had been added. The number of tiles in the training set increased between the start and the end of the data-gathering period.

Diagnostic class	Training tiles (512 × 512)	
	At start	At end
Adenocarcinoma	1130	1130
Adenoma	898	939
Anal mucosa	67	67
Artifact	361	463
Connective tissue	162	165
Hyperplastic polyp/sessile serrated lesion	231/22	240/22
Immunohistochemistry	61	61
Inflammation	612	618
Normal large bowel	1158	1197
Normal small bowel	309	310
Total	4758	4950

### Training and optimization

The model was trained using the image classification tools in the Fast.ai library,^6^ which itself is built on PyTorch.^7^ The tiles forming the dataset had been classified by a consultant pathologist first, as above. The Fast.ai library includes models that have been pre-trained on the ImageNet^8^ dataset for transfer learning purposes so the model training focused on fine-tuning the network “head” weights with the backbone of the model weights frozen. An investigation into fine-tuning the whole model offered no accuracy improvements. A ResNet50^9^ model was identified as offering the best accuracy out of the class of ResNet models, with the larger ResNet101 model not offering any significant improvement with the trade-off of increased training and inference latency. Twenty-five per cent of the tiles were randomly selected as a validation set and used for identifying early-stopping of the network training. Images were augmented using random vertical and horizontal flipping during training.^10^ The default optimizer and learning rates in the Fast.ai library were used.^11^ An investigation into using different learning rates, including those recommended by the learning rate finder in Fast.ai, offered no significant improvement. An AWS P3 instance was used with a single V100 GPU for training. The model converged within 50 epochs over the training data.

### Dominant diagnosis

On receiving the tile classifications with probability scores from the AWS virtual machine, the Free D Path software calculated a dominant diagnosis for that tile. Firstly, tiles with a classification probability score of less than 0.75 were excluded. The classifications of the remaining tiles were then used to calculate a dominant diagnosis for the slide according to the following hierarchy. The dominant diagnosis for the slide being decided by the highest ranked tile diagnosis:

• Adenocarcinoma
• Adenoma
• Anal mucosa
• Hyperplastic polyp/sessile serrated lesion (HP/SSL)
• Inflammation
• Normal small bowel
• Artifact
• Immunohistochemistry
• Normal large bowel.

Each biopsy specimen could have several slides and the highest ranked slide-level diagnosis was then used as the specimen-level diagnosis and then the highest ranked the specimen-level diagnosis was used as the case-level diagnosis. So, for example, if one tile in any of slides was classified as “adenocarcinoma” with a probability of greater than 0.75, then the case would be classified as “adenocarcinoma” for the purpose of triaging the case.

### Turnaround times

The reporting turnaround times were calculated by subtracting the timestamp at which the scanning of the case was completed from the timestamp of the report being authorized. The first of these timestamps was extracted from the Cerebro laboratory management platform and the second time stamp was from the reporting platform.

Our laboratory did not have a permanent backlog of large bowel biopsies, but we chose a period in the spring of 2022 and another in the summer of 2022 to gather our performance data. These were both periods in which annual leave arrangements had resulted in a temporary shortage of pathologists, and a reporting backlog of several days.

## Results

The automated download of a WSI from the Philips scanner platform took about 100 s per slide and then another 75 s to send the WSI to the virtual machine and receive a diagnostic classification back; a total of approximately 175 s per slide. Slides with a lot of tissue took longer as the WSI files were larger. The WSIs had been downscaled and partly cropped on the scanner platform prior to download and were approximately 15 MB each.

The study was conducted mainly by NHS staff within the scope of their employment and so there were no staff salary costs specifically attributable to this project. Project expenses amounted to £14800, including £8500 for developing the robotic process automation solution to download WSIs and £1800 for Amazon Web Services hosting charges for approximately three years of use.

**Fig. 3:**
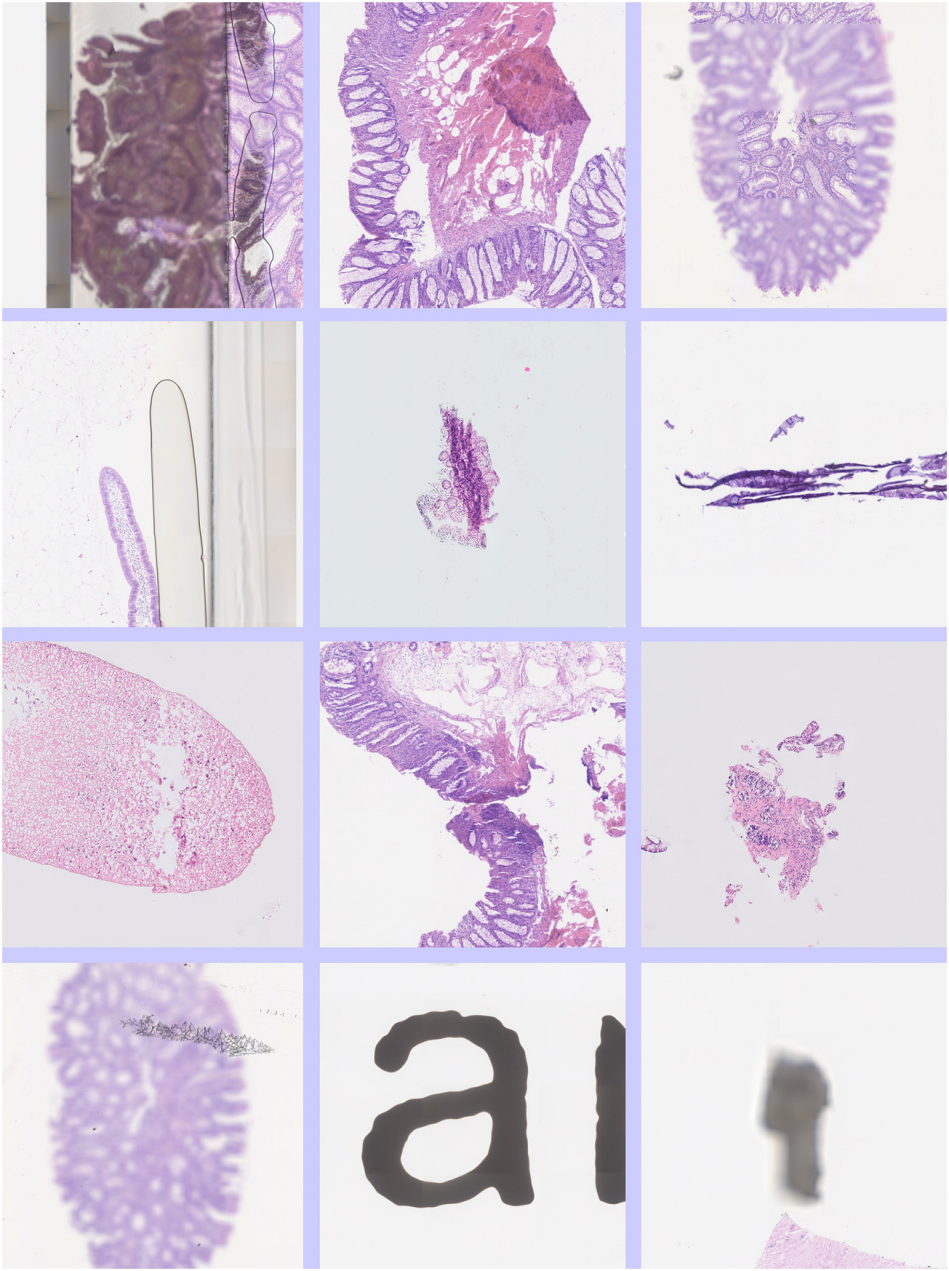
Tiles showing various forms of artifact. From top left (column, row): (1,1) A misaligned coverslip. (2,1) Folded tissue and diathermy artifact. (3,1) Tissue partly out of focus. (1,2) An air bubble in the coverslip mountant. (2,2) & (3,2) Folded tissue. (1,3) Fecal material. (2,3) & (3,3) Diathermy artifact. (1,4) Scratch on coverslip and loss of focus. (2,4) Printing on slide. (3,4) Dust on coverslip.

Fig. 3 shows a variety of artifacts in the image tiles. Most WSIs showed at least one tile with some artifact. The most common was a coverslip edge or printing on the slide.

**Fig. 4:**
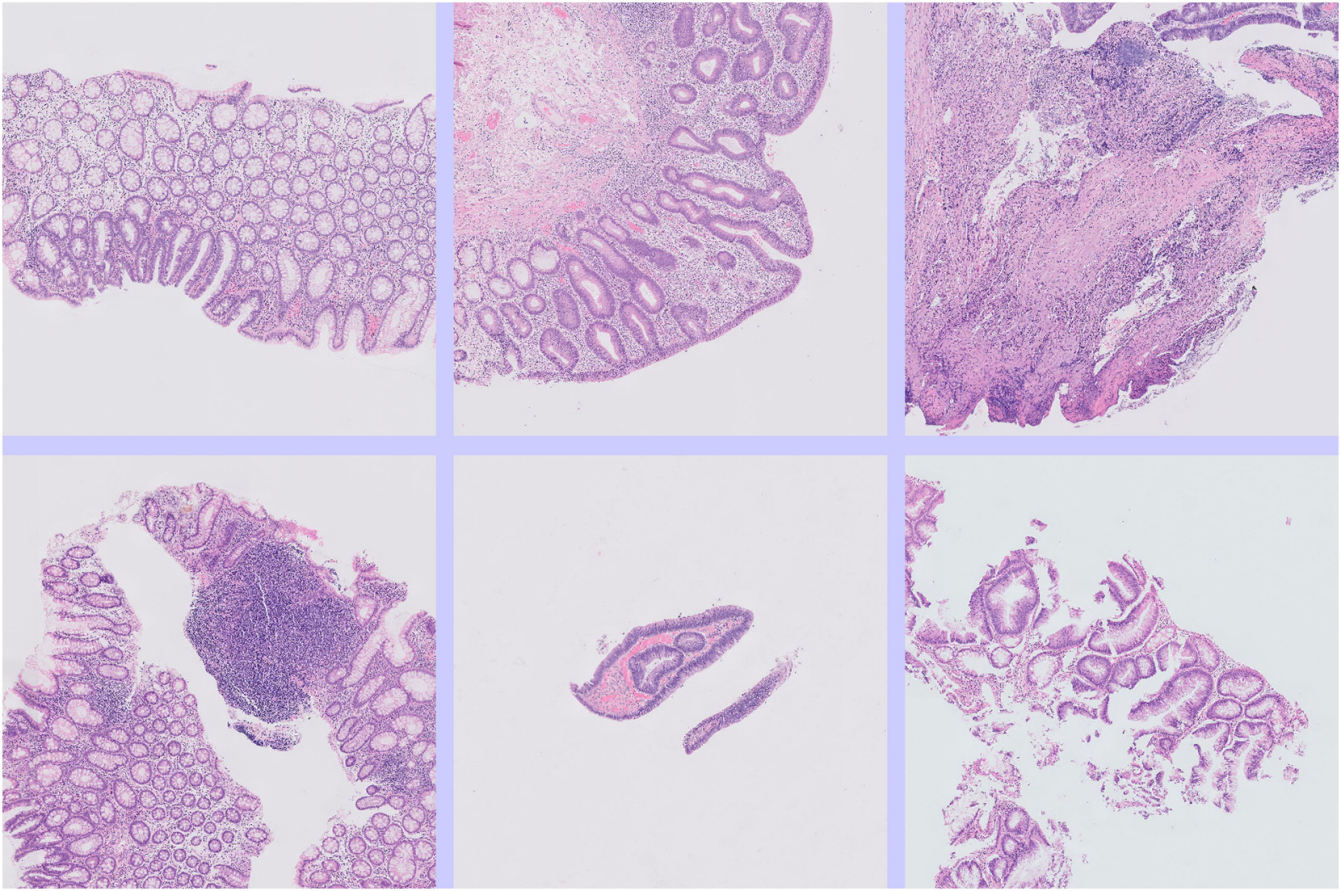
Tiles from adenomas that were mislabeled by the AI as non-neoplastic. This error was often related to there being relatively little neoplastic tissue in the image.

Fig. 4 shows some tiles that were truly from adenomas but were mislabeled by the AI as some non-neoplastic class of diagnosis. This was often because there was only a small amount of neoplastic tissue in the tile. This error did not usually lead to an error in the slide-level diagnosis, as one or more other tiles from the WSI were correctly labeled.

**Fig. 5:**
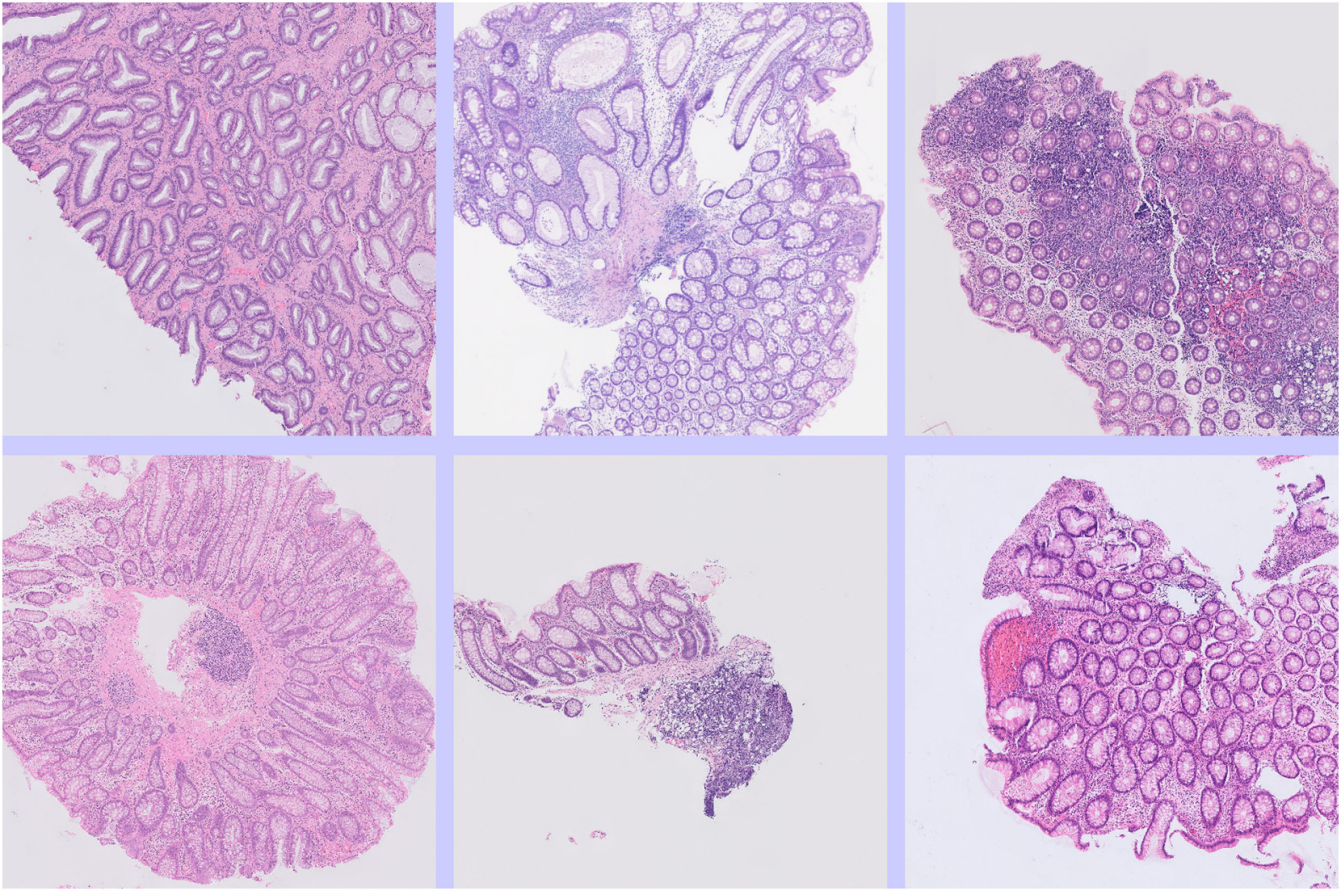
Tiles from non-neoplastic large bowel that was mislabeled by the AI as neoplastic (adenoma). This error was often related to architectural distortion of the tissue, or suboptimal plane of sectioning.

Fig. 5 shows some tiles that were mislabeled by the AI as showing adenoma when they were truly non-neoplastic. These often showed some degree of distortion of the crypt architecture.

**Fig. 6:**
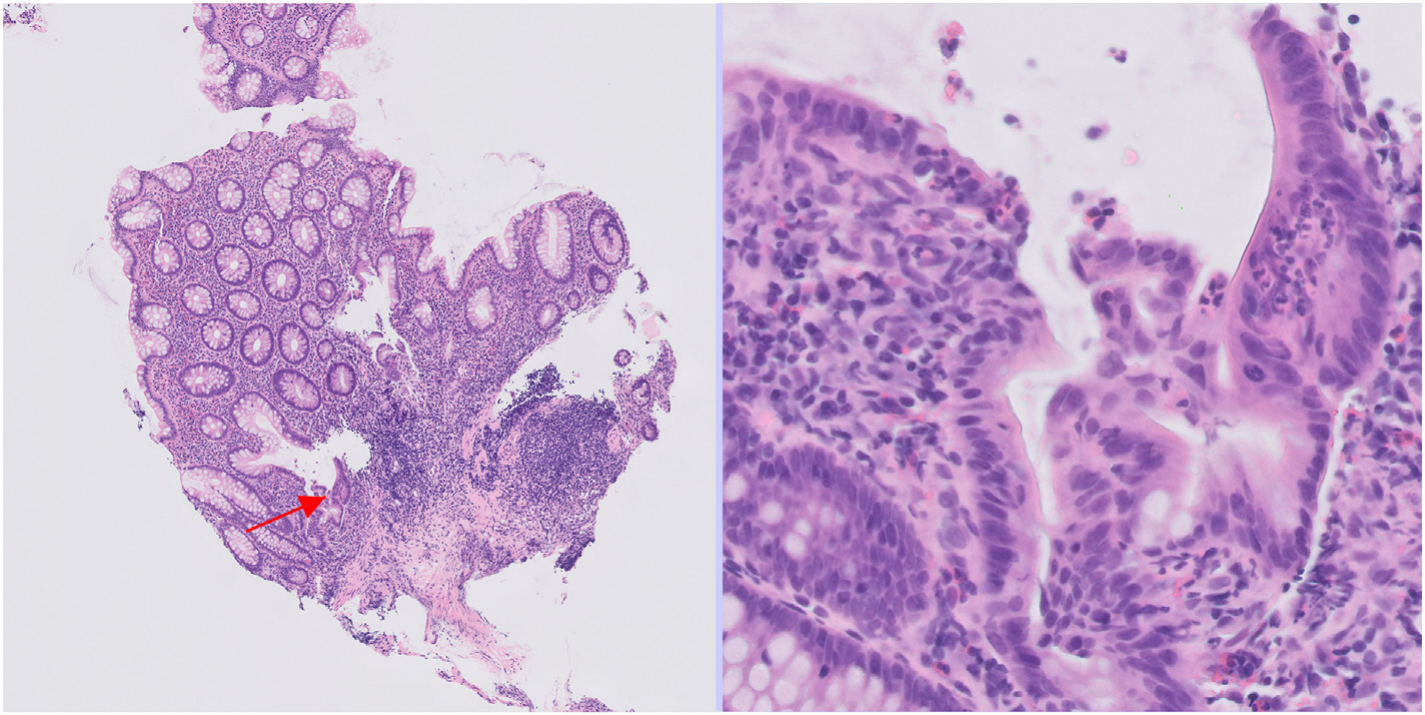
A tile that was mislabeled by the AI as normal large bowel but showed a focal neutrophilic cryptitis (arrowed) on pathologist review.

**Fig. 7:**
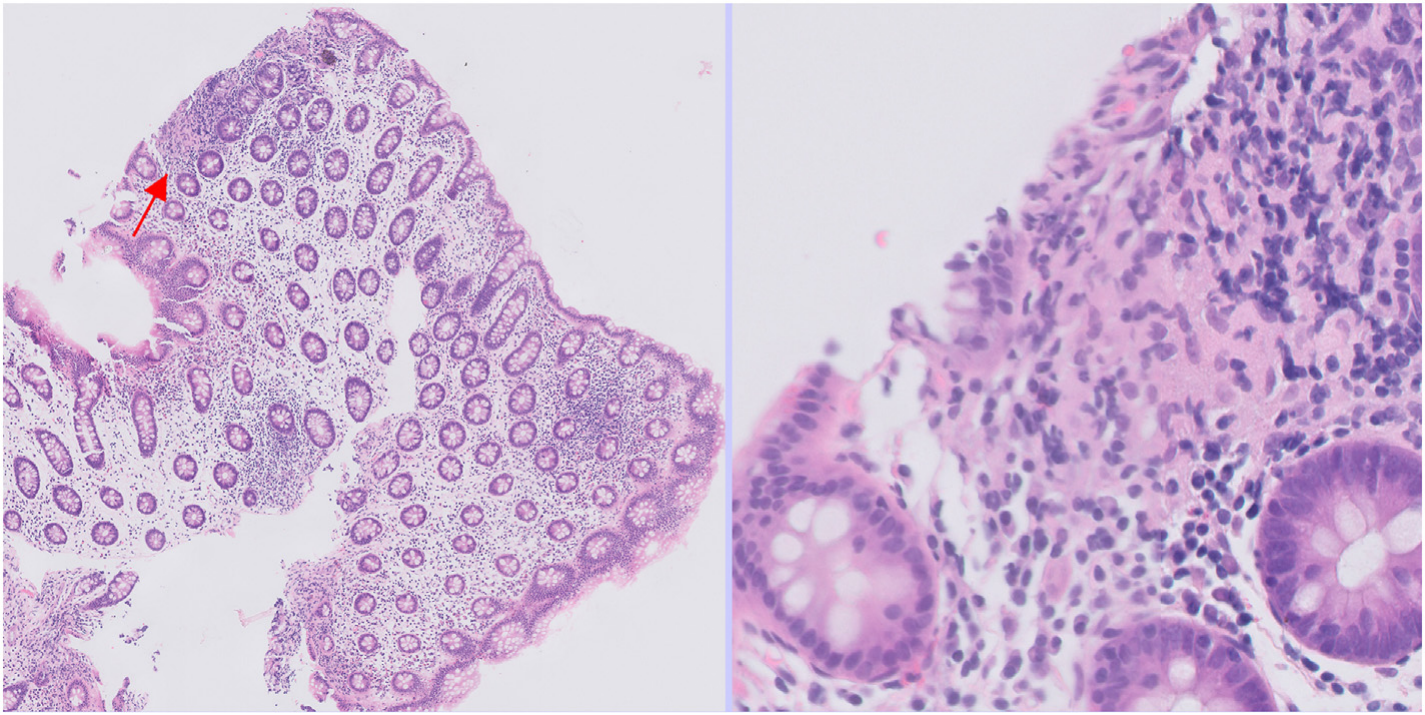
A tile that was mislabeled by the AI as normal large bowel but showed a microgranuloma (arrowed) on pathologist review.

Fig. 6, Fig. 7 show slides that were misclassified as normal large bowel when they truly showed focal inflammatory changes that were only obvious on high-power magnification.

**Fig. 8:**
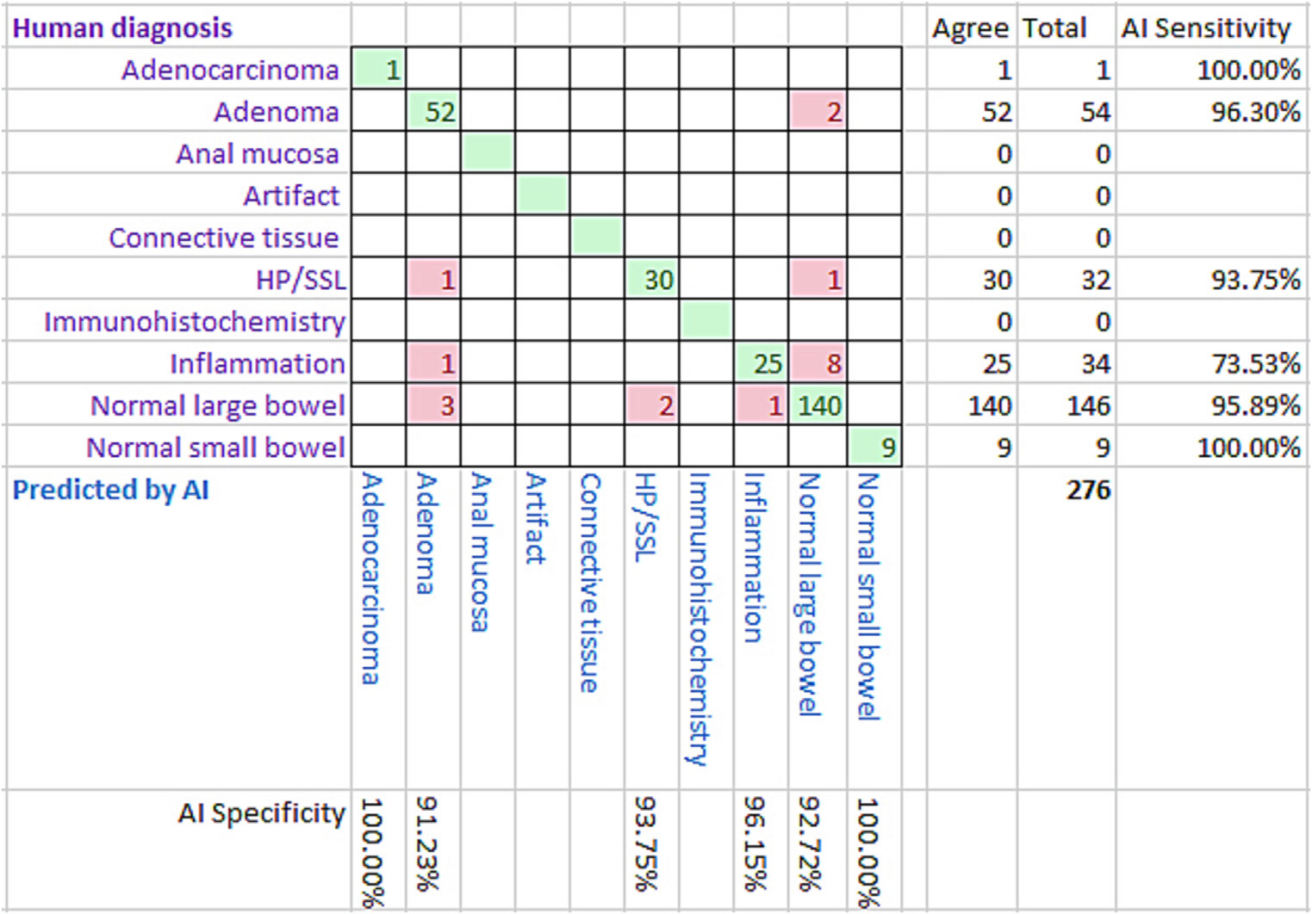
A confusion matrix displaying specimen-level diagnoses.

**Fig. 9:**
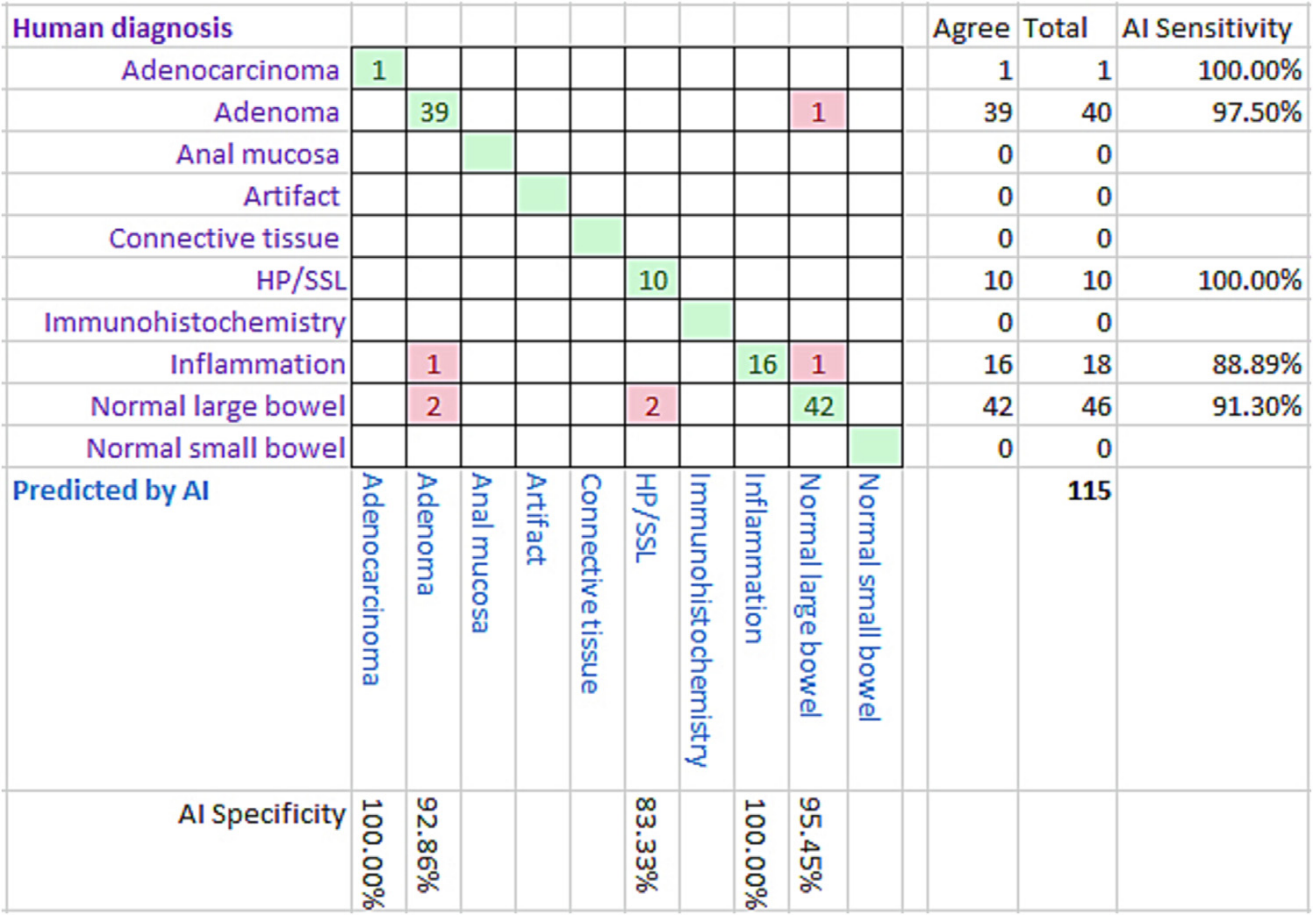
A confusion matrix displaying case-level diagnoses.

The results for the specimen-level diagnoses are set out in Fig. 8 and the results for the case-level diagnoses are set out in Fig. 9.

**Fig. 10:**
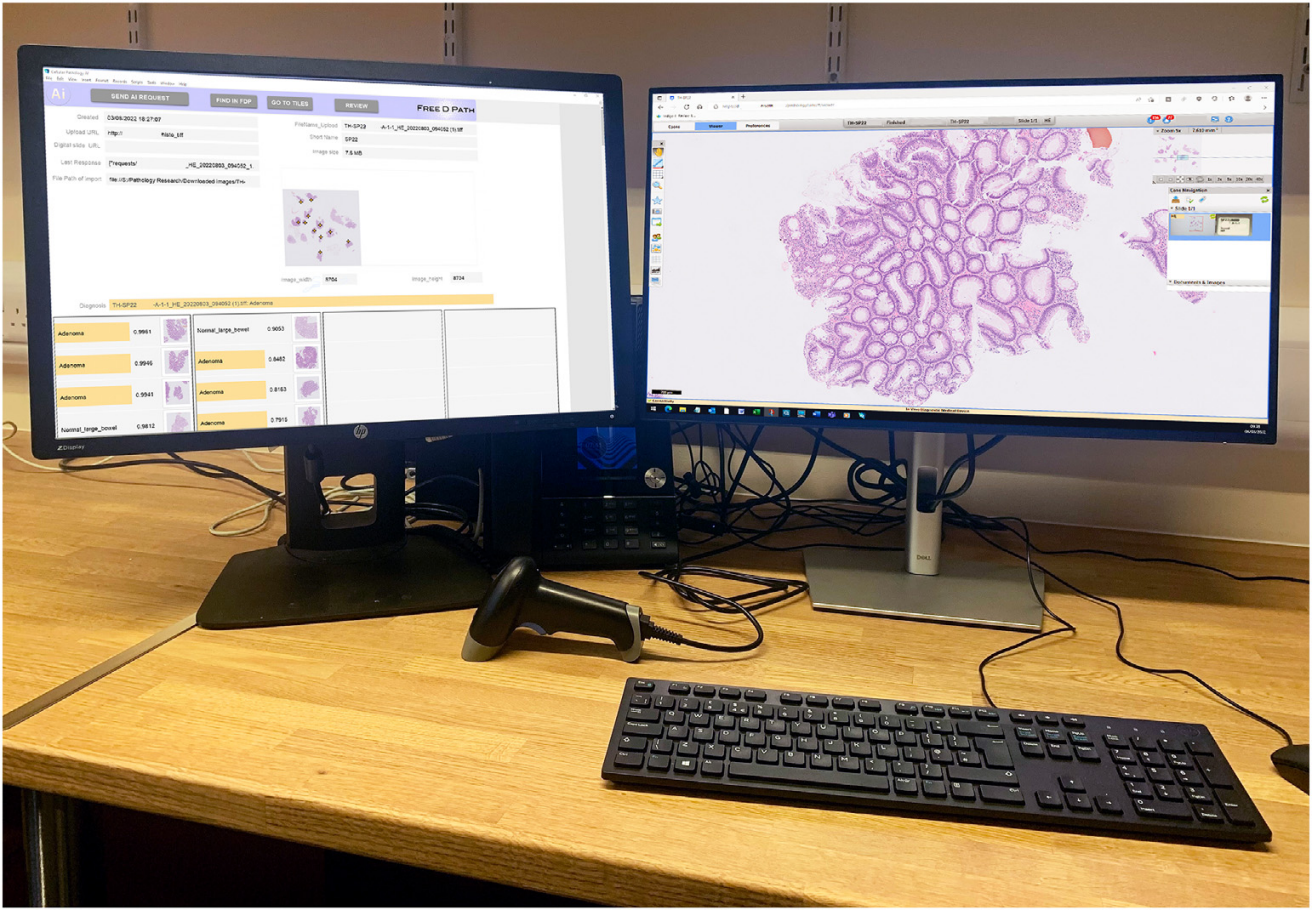
A reporting workstation with a digital slide displayed on one monitor (right) and the corresponding image tiles along with AI diagnostic labels and image map displayed on the other monitor. The amber conditional color formatting warns the pathologist that the AI has detected a neoplasm. Case identifiers have been hidden in the image.

Fig. 10 shows a reporting workstation with a digital slide displayed on one monitor (right) and the corresponding image tiles along with AI diagnostic labels and image map displayed on the other monitor. While reporting the case, the pathologist could correct any tiles that had been mislabeled by the AI.

Table 1 shows the number of 512 × 512 pixel tiles in the training set for each diagnostic class. These increased during the study as mislabeled slides were corrected by the reporting pathologist and added.

Table 2 shows the sensitivity and specificity for various combinations of specimen-level diagnosis and case-level diagnosis. The case-level diagnosis was used to prioritize the cases for reporting.

**Table 2:** Sensitivity and specificity for various combinations of specimen-level diagnosis and case-level diagnosis (HP/SSL = hyperplastic polyp and sessile serrated lesion).

AI diagnosis	Specimen-level		Case-level	
	Sensitivity	Specificity	Sensitivity	Specificity
Adenomas and adenocarcinomas	96.36%	91.38%	97.56%	93.02%
Adenomas, adenocarcinomas and inflammation	87.64%	92.86%	94.92%	94.92%
Adenomas, adenocarcinomas, inflammation and HP/SSL	89.26%	93.10%	95.65%	92.96%
Normal small bowel or large bowel	96.13%	93.13%	91.30%	95.45%

Table 3 shows that reporting became quicker for cases with a neoplastic lesion or inflammation when AI triage was used. The AI triage pathway did not prioritize other classes of diagnosis for reporting and these specimens showed no difference in reporting times for the routine (non-AI) pathway and the AI pathway.

**Table 3:** Mean turnaround times from the completion of scanning to report authorization, with and without triage by AI. Times are given in days and hours (dd:hh).

	Routine pathway		AI triage pathway		
Diagnosis	Cases (n)	dd:hh	Case (n)	dd:hh	Two tailed T-test: P value
All neoplasia	47	05:20	41	02:17	0.00000009
Adenocarcinoma	2	12:21	1	01:17	Too few cases
Adenoma	45	05:13	40	02:18	0.00000003
Inflammation	17	05:23	18	03:05	0.04628485
HP/SSL	13	05:00	10	04:07	0.31885666
Normal small or large bowel	26	05:07	46	04:15	0.33809500

## Conclusions

This project showed that NHS laboratory staff with no prior machine learning experience could design and implement an AI solution to accurately triage large bowel biopsies into several diagnostic classes and this improved reporting turnaround times for biopsies with neoplasia or inflammation. There was some remote expert assistance.

AI is a fast-moving field in which notable advances are rapidly superseded. There has been a recent (2021) previous description of an AI-based pre-screening tool capable of identifying normal and neoplastic colon biopsies^3^ but this only classified the biopsies into two diagnostic classes, neoplastic and non-neoplastic, and was not integrated into a real-world cellular pathology reporting pathway or used for pathway management. This previously described pathway is interesting in that it used weakly supervised learning, in which the slide-level diagnostic label was used for training. In contrast, our study divided the WSI into small tiles and a pathologist then verified a diagnostic label for each tile. The former approach requires less pathologist time but the latter approach is more efficient in that fewer digital slides are needed to obtain an accurate classifier. In some scenarios, one might expect the tile-level labeling to be up to 100 times more efficient but in the context of bowel biopsies the difference is probably not so great, as typically the amount of tissue on the slide is only small and the whole image would only generate a few tiles. Slide-level labeling works best when all the tissue on the slide is uniform, so that the slide-level label will be accurate for all daughter tiles. Although it was quite common for biopsies to show several types of tissue, for example normal large bowel, an adenoma, and inflammation in the same biopsy, the small size of the biopsies tends to make this less likely than it would be for large pieces of tissue. In this previous study, WSIs generating fewer than four tiles were excluded. Excluding slides was not appropriate in our study, as we were interested in a successful triage strategy and prioritized sensitivity over specificity. This previous study applied some color normalization during processing of the images. This can be useful when the images are from different scanners or the slides are from different laboratories with different staining protocols but can give unexpected results if there is only a small amount of tissue in the image. In our study, all the slides were from our laboratory and scanned on the same scanner. We did not need to use any color normalization.

Another paper from 2021 on large bowel pathology also used the whole slide labels for weakly supervised learning but with a particularly large training dataset of  42655 “patches” or tiles (300 × 300 pixels) from 559 slides at ×20 magnification.^2^ By contrast, our study used a training set of up to 4950 tiles (512 × 512 pixels) from approximately 220 slides and at ×5 magnification (see Table 1). The optimal level of magnification for tiles depends on the specimen type and differential diagnoses. Pathologists tend to report large bowel biopsies using mainly low power magnification. This allows the architecture of the tissue and the interrelationship of features to be understood. However, some features, such as neutrophilic cryptitis (Fig. 6) or microgranulomas (Fig. 7), are difficult to see at ×5 magnification. Using a higher magnification gives more cellular detail in the image but less information about the overall architecture of the tissue. The way forward may be a combination of low- and high-power examination, but this would require increased computational resources.

As explained above, active learning^12^ was used to improve the performance of the AI. The reinforcement of the initial training set was through the addition of tiles that had been mislabeled by the AI and relabeled by a pathologist. A pathologist reviewed approximately  10000 tiles during this reinforcement of the training set, but most of these had been labeled correctly by the AI and so were not added to the training set.

Fig. 8 displays the accuracy of the AI specimen-level classifications versus the “ground-truth” human pathologist classifications. As explained above, the slide-level classification was used to determine a case-level classification, and this was used to decide the priority of the case for reporting. Fig. 9 gives the same information for case-level diagnoses. Table 2 gives the sensitivity and specificity for various aggregates of diagnostic cases. In our study, any case that showed an adenoma, adenocarcinoma, or inflammation was prioritized for reporting using the case-level diagnosis, with a sensitivity of 94.92% and specificity also of 94.92%. Previous studies have focused on slide-level diagnoses and with a more limited range of diagnostic classes, often just neoplastic and non-neoplastic. In our study, neoplastic slide-level diagnoses (adenoma and adenocarcinoma) had a specificity of 96.36% and a sensitivity of 91.38%. This is similar to the approximately equivalent metrics in a recent previous study.^3^ A direct comparison is not possible as we only used small biopsy specimens from a reporting backlog, but other studies of AI diagnosis of large bowel neoplasia have included a higher proportion of adenocarcinomas,^3^ bowel excision specimens and even frozen section material.^2^ The AI platform was trained to recognize all the diagnostic classes listed above in the method, but some of these were never used as a slide- and case-level diagnosis. For “artifact”, this was because tiles with this label were excluded as explained above. The other unused diagnostic classifications, “anal mucosa”, “connective tissue”, and “immunohistochemistry”, never occurred as a dominant slide-level diagnosis.

The AI triage pathway prioritized neoplastic diagnoses (adenocarcinoma and adenoma) and inflammation for reporting and showed significantly reduced reporting times for these cases, particularly for neoplastic cases, as set out in Table 3. The cases with other AI diagnostic classifications were not prioritized for reporting and had similar reporting times to routine work. The neoplastic cases were almost all adenomas, as adenocarcinomas were usually clinically obvious at endoscopy and the associated biopsies did not accrue as backlog work.

In this study, we used AI to improve workflow but the final diagnosis was made by a human pathologist. However, the AI’s level of accuracy seems to suggest that it could perform at a similar level to a human in the interpretation of WSIs. A WSI is not perfect representation of a glass slide and there are a range of issues, such as downgraded resolution and variable precision of focus, that tend to make diagnoses from WSI less accurate than for glass slides.^13^ There have been numerous studies comparing the concordance of WSI diagnoses and glass slide diagnoses. In a review of studies of WSI reporting versus glass slide reporting that emulated a real-world clinical environment, the accuracy of diagnosis was 89% for WSIs and 92% for glass slides.^14^ In this review, the concordance between WSI and glass diagnoses was 86%, but improved to 98% if minor discordance was accepted. In our study, there was concordance in 257/276 (93%) for specimen-level AI interpretation of WSI versus human interpretation of glass slides. Some of the errors made by the AI were errors that you might expect a human to make. Missing a small focus of neutrophilic cryptitis (Fig. 6) or a microgranuloma (Fig. 7) are common human pathologist errors. However, the AI also made some errors that would be difficult for a human pathologist to understand. Fig. 5 shows several examples of non-neoplastic mucosa that were mislabeled as being adenomatous in a way that a human would be unlikely to replicate. In addition the AI classifier had a limited diagnostic range. If a biopsy in our study had contained lymphoma or amyloid, then it would have been impossible for the AI to correctly label it, as it has never been trained to recognize these conditions. There are many rare conditions in large bowel pathology and so it is quite common for a biopsy to show a rare condition. Humans are more efficient learners than AI platforms and can learn to recognize a new entity after seeing only one or two examples while an AI platform requires training on many examples.^15^ This makes it logistically difficult to train an AI platform to recognize a wide range of rare diagnoses. The reduced efficiency of weakly supervised learning is discussed above, and while weakly supervised learning might have some advantages for common diagnoses with almost unlimited training material, it would be a less appropriate strategy for rare diagnoses with limited training material. AI can be particularly effective when applied to a narrowly defined task for which it can be intensely trained, such as counting or scoring one or more specific features. Gleason scoring of prostatic carcinoma is an example of this,^16^ as in the assessment of donor kidneys prior to implantation.^17^ In the latter two roles, AI was used to assist pathologists in improving performance, as it was in our study. We are still some way from AI being able to make diagnoses without human supervision and this is partly because training AI to interpret a wide range of rare scenarios is difficult.

In conclusion, this service evaluation study was successful at triaging large bowel and biopsies showing neoplasia or inflammation were reported more quickly. It is also quite an affordable and feasible approach for larger laboratories with IT-savvy staff. The mean reporting turnaround time for all cases is unlikely to change greatly. The method does not reduce the pathologists’ overall workload and the fundamental workload demand/pathologist resource mismatch remains unresolved.

## Positive points

• Open-source AI image classification solutions have become easy and inexpensive to use.
• NHS laboratory staff with no prior experience of AI were able to design and implement an AI solution to accurately triage large bowel biopsies into several diagnostic classes and this improved reporting turnaround times for biopsies with neoplasia or inflammation.
• Laboratory-developed software allowed the AI solution to be interfaced with the digital slide scanner platform and the cellular pathology platform to allow the pathway to be automated.

## Negative points

• Interfacing AI platforms with digital slide scanners and cellular pathology reporting platforms was difficult and required substantially more resources than the development and operation of the AI solution itself.
• The advantage of this approach is only significant in a situation in which there is a reporting backlog.
• The method is specific to the laboratory where the study took place and is not easily transferable to another laboratory.
• The method requires that the laboratory can digitally scan slides, but this capability is not currently widespread.

## Competing interests

Frederick Mayall, Louis de Mendonça, Sarah Brownlie and Azka Anees are employees of Somerset NHS Foundation Trust. Frederick Mayall and Stephen Perring are directors and shareholders of Diagnostic Path Solutions Limited, an IT company that is part owned by the Taunton and Somerset NHS Foundation Trust. Mark Goodhead is a director of Longshot Systems, an IT company with an interest in trading software.

## Author contributions

**Study concept and design:** Mayall, Goodhead, Perring. **Funding obtained:** Mayall. **Conduct of the study and method development:** Mayall, de Mendonça, Brownlie, Anees, Goodhead, Perring. **Analysis and interpretation of data:** Mayall, de Mendonça, Goodhead. **Drafting of the manuscript:** Mayall. **Critical revision of the manuscript for important intellectual content:** Mayall, de Mendonça, Brownlie, Anees, Goodhead, Perring. **Study supervision:** Mayall, Perring.
